# Sulcal Cavitation in Linear Head Acceleration: Possible Correlation With Chronic Traumatic Encephalopathy

**DOI:** 10.3389/fneur.2022.832370

**Published:** 2022-02-28

**Authors:** Joseph Kerwin, Atacan Yücesoy, Suhas Vidhate, Bianca M. Dávila-Montero, Jacob L. Van Orman, Thomas J. Pence, Michaelann Tartis, Ricardo Mejía-Alvarez, Adam M. Willis

**Affiliations:** ^1^Department of Mechanical Engineering, Michigan State University, East Lansing, MI, United States; ^2^Department of Neurology, Brooke Army Medical Center, Fort Sam Houston, TX, United States; ^3^Department of Chemical Engineering, New Mexico Institute of Mining and Technology, Socorro, NM, United States

**Keywords:** cavitation, mild traumatic brain injury, blunt impact, drop tower, high-speed imaging, brain phantoms, chronic traumatic encephalopathy

## Abstract

Traumatic Brain Injury (TBI) is a significant public health and financial concern that is affecting tens of thousands of people in the United States annually. There were over a million hospital visits related to TBI in 2017. Along with immediate and short-term morbidity from TBI, chronic traumatic encephalopathy (CTE) can have life-altering, chronic morbidity, yet the direct linkage of how head impacts lead to this pathology remains unknown. A possible clue is that chronic traumatic encephalopathy appears to initiate in the depths of the sulci. The purpose of this study was to isolate the injury mechanism/s associated with blunt force impact events. To this end, drop tower experiments were performed on a human head phantom. Our phantom was fabricated into a three-dimensional extruded ellipsoid geometry made out of Polyacrylamide gelatin that incorporated gyri-sulci interaction. The phantom was assembled into a polylactic acid 3D-printed skull, surrounded with deionized water, and enclosed between two optical windows. The phantom received repetitive low-force impacts on the order of magnitude of an average boxing punch. Intracranial pressure profiles were recorded in conjunction with high-speed imaging, 25 k frames-per-second. Cavitation was observed in all trials. *Cavitation* is the spontaneous formation of vapor bubbles in the liquid phase resulting from a pressure drop that reaches the vapor pressure of the liquid. The observed cavitation was predominately located in the contrecoup during negative pressure phases of local intracranial pressure. To further investigate the cavitation interaction with the brain tissue phantom, a 2D plane strain computational model was built to simulate the deformation of gyrated tissue as a result from the initiation of cavitation bubbles seen in the phantom experiments. These computational experiments demonstrated a focusing of strain at the depths of the sulci from bubble expansion. Our results add further evidence that mechanical interactions could contribute to the development of chronic traumatic encephalopathy and also that fluid cavitation may play a role in this interaction.

## 1. Introduction

The consequences of traumatic brain injury (TBI) can be both immediate (loss of consciousness, amnesia, headaches, disequilibrium, intracranial hemorrhage, etc.) and subacute (post concussive syndrome (PCS), post traumatic stress disorder (PTSD), etc.) Additionally, this injury can also initiate a progressive neurological decline, Chronic Traumatic Encephalopathy (CTE). CTE has a distinct neuropathological progression (tauopathy), appearing to initiate within the depths of sulci and perivascular location (1), as is found in boxing and American football athletes (2, 3). CTE is associated with repetitive head impacts and there exist a dose-response between the occurrence and severity of CTE with the duration of participation in impact sports (4). The localization of pathology and its initiation within the sulci is also supported by human imaging studies (5) of soccer players showing increase sulcal volume after single season of play.

One possible physical mechanism that localizes to the depths of the sulci is strain / strain rate during brain motion following impact. Multiple computational models have predicted that brain deformation would have local maxima of strain / strain rate within and at the depths of the sulci (6, 7). Both utilized computer analysis that factored for human brain anatomy and tissue properties to predict the structural response of brain material to different kinds of head impact. In particular, computational simulations demonstrated that the area of greatest tissue deformation lies in the depths of the sulci, deep grooves that separate folds of brain. (7) also predicted cavitation to occur near skull / brain interface and to a lesser extent near the CSF brain interface. In conjunction with sulcal strain focusing from gyral deformation, other investigators have suggested more complex interaction of the cerebral spinal fluid (CSF) during impact, such as the “water hammer” effect (5). However, current computational models of TBI have not explicitly modeled CSF dynamics with dedicated hydrodynamics simulations coupled with compliant boundaries, and as such, are not able to fully resolve the interplay of CSF and adjacent brain material during impulsive loading. Thus, a further refinement of (5) “water hammer” effect is the resultant complex dynamics of cavitation bubbles and cerebral tissue. However, fluid cavitation in head trauma has been controversial.

The controversy of fluid cavitation during impact extends from the lack of direct evidence present for cavitation within intracranial contents. Cavitation is a phenomenon observed in liquid environments when the hydrostatic pressure falls below a critically low threshold resulting in the nucleation and expansion of vapor bubbles. The phenomenon has been investigated for over a century (8) and is found in dynamic fluid flows (9) or impulsive loading upon fluid filled containers (10) when local pressures fall near vapor pressure (for water / CSF this is below -100 kPa at STP) (11). Within mechanical systems, damage from the effects of cavitation could occur from multiple mechanisms. For example, following bubble nucleation and expansion, these bubbles can collapse and produce very high pressures and temperatures which could affect adjacent structures (9). Furthermore, if a bubble collapses occurs near a surface, a high speed microjet is formed and directed toward the surface (12, 13), leading to surface damage. It has been shown that microjetting can cause significant damage to propellers and other machinery (14–16) and thus if occurring during head trauma, could be a hypothesized mechanism by which cavitation causes brain damage. More specifically, if cavitation were to occur near a soft material such as human biological tissue, the production of high local strain from material displacement to accommodate an adjacent bubble expansion may also lead to tissue damage (17).

Cavitation was first suggested as early as 1948 (18) as a direct injury mechanism for traumatic brain injury (TBI) and further refined in the late 1950's (10). (10) introduced the concept of cavitation as a way to explain the mechanism of contrecoup contusions found in blunt head injury. Namely, the force of a focused head impact produces a drop in CSF hydrostatic pressure over the contrecoup site caused by a difference in the inertia between the skull and enclosed brain.

Such a mechanism intuitively explained the contrecoup injury found in the frontotemporal regions following occipital impacts (10, 19). However, skepticism arose because it was difficult to reconcile this mechanism with the fact that frontal impacts did not usually lead to occipital contusions (20–22). As a consequence, cavitation remains controversial as an injury mechanism in TBI, and by the 1970s cavitation as a mechanism of contusion was replaced by the hypothesis that tissue injury following trauma was secondary to the brain impacting or sliding against the skull (20–22).

As has been highlighted in the blast TBI literature, skull shape and size can greatly modulate the transmission of stress intracranially during impact. This phenomenon has been addressed experimentally by choosing an appropriately skull-based scaled animal model (23). More recently, by directly modeling blast interactions with human-based skulls, computational engineering analysis has greatly improved our understanding of TBI and predicted the inception of cavitation (24). Cavitation is suggested as a mechanism of injury in blast related TBI. This has been demonstrated by computational analysis (25–28) and observed experimentally using models of the human head (29, 30). Computational analysis has also predicted cavitation to occur in blunt TBI (25, 31). Although, the onset of cavitation has been predicted by computer models, the interaction of cavitation bubbles with brain tissues still remains unresolved (computationally) as does the expected and the anatomic distribution of cavitation bubbles during impulsive loading. Thus, a clear association of cavitation with a specific human neuropathology is still lacking.

Although computational methods are limited in their resolution of cavitation physics in head trauma, experimental visualization of cavitation bubbles near soft surfaces by (17) quantified the interaction of bubble dynamics with soft materials and demonstrated bubble expansion produced greater adjacent tissue strain than bubble collapse. However, the simplistic geometry of these experiments limit the conclusions that can be drawn in association for TBI in a gyrated human head. Recently, a more complex human skull / gelatin brain phantom showed intracranial cavitation (30), and quantification of brain motion, however this effort lacked the resolution to generalize how these bubbles would interact with anatomic regions of interest. The challenge remains of developing a tissue phantom which contains enough realism to provide insight into human pathology and quantify tissue / CSF dynamics in experiments which recapitulate the intracranial mechanical loading during head trauma.

To this end, we aim to experimentally investigate:

1) if cavitation occurs in realistic head surrogates undergoing blunt impact,
2) analyze tissue response / interaction with the expanding cavitation bubbles,
3) and determine if the loading / deformation patterns associated with cavitation correlate to known neuropathology.

## 2. Materials and Methods

### 2.1. Brain Phantom Design and Fabrication

**Figure 1:**
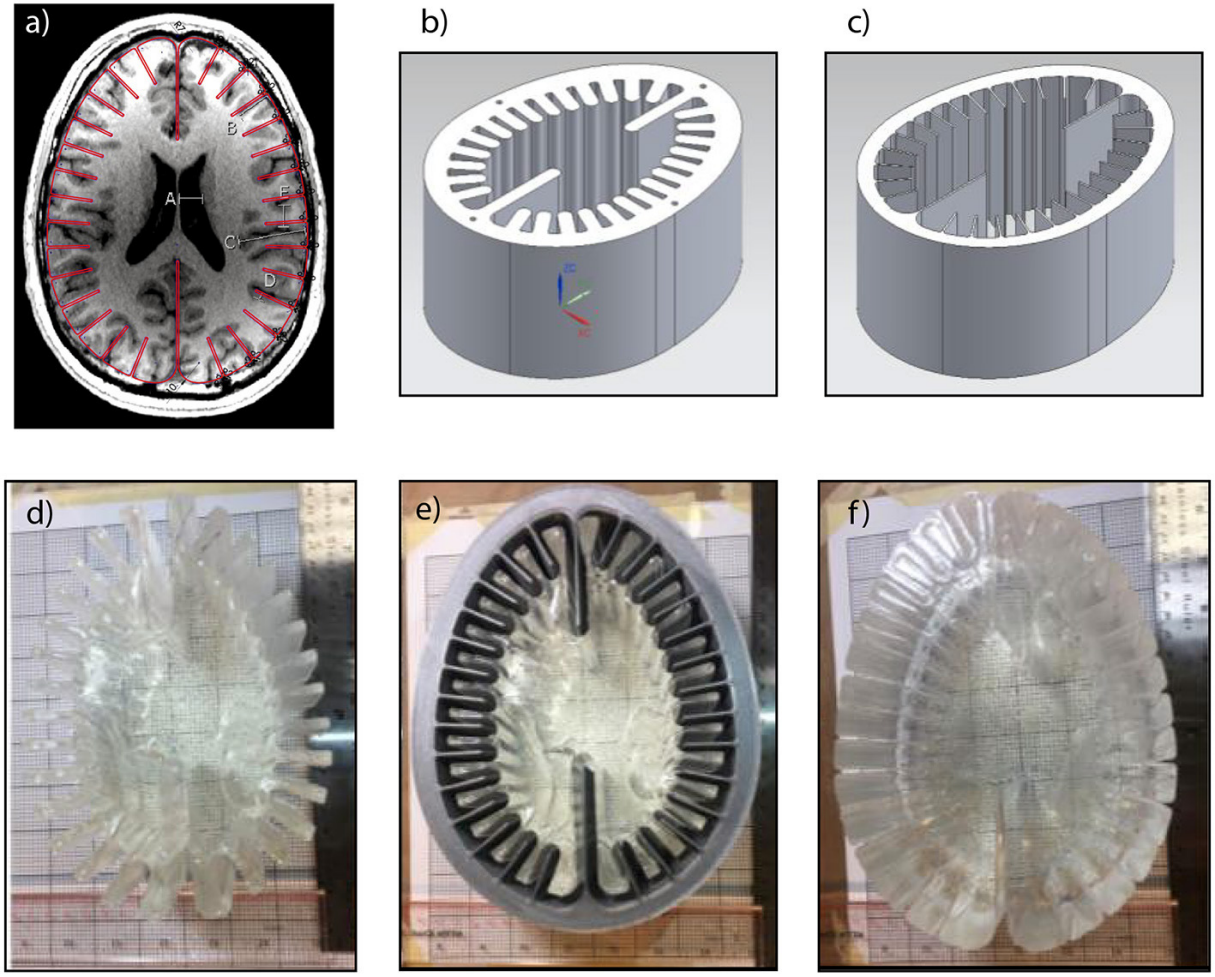
Fabrication process of simplified Biofidelic Brain Phantom; **(a)** MRI image of axial cut of principle investigator's brain with simplified outline (red line); **(b)** mold for layer 1; **(c)** mold for layer 2; **(d)** fabricated layer 1; **(e)** layer 1 inside layer 2 mold; **(f)** fabricated two-material brain phantom.

This study is conducted with a simplified biofidelic phantom of the human brain. As shown in Figure 1a, this phantom grossly represents the axial anatomy of a human head / brain. The design captured only dominant geometrical dimensions of the sulci, gyri, gray matter thickness, and overall brain dimensions. Some elements of the full phantom design, such as ventricles and vasculature, were omitted for this study. Figure 1f shows the resulting gelatin-based two-material brain phantom. In the mid-plane of our test object, a thin layer of micron-sized particles were distributed for use with particle image velocimetry algorithms, which could resolve local deformation.

The biofidelic brain phantom was created using different weight percent concentrations of Polyacrylamide (PAA) gelatin. PAA is an ideal material for a brain tissue simulant due to its easily tunable material properties, room temperature fabrication, and high level of transparency for optical access. Additionally, PAA has a relatively short assembly time and low energy requirements making it a superior choice over other common inorganic gelatin. PAA results from the cross-linking of two monomers, Acrylamide and Methylene-bis-Acrylamide (MBA). Ammonium Persulfate (APS) induces these two monomers to cross-link, and the process is catalyzed with N,N,N',N'-tetramethylethylenediamine (TEMED). The proportion of the components was tailored to approximate the bulk mechanical properties of white and gray matter (32).

To generate the inner layer (Layer 1) simulant, 83 g of Acrylamide (purity ≥98%, gas chromatography, Sigma-Aldrich, USA) were dissolved in 830 ml of deionized (DI) water, resulting in a 10% (weight / volume) solution. When the Acrylamide was fully dissolved, 2.77 g of *N,N'*-methylenebis(acrylamide) (MBA, purity 99%, Sigma-Aldrich) was slowly added to the solution while stirring and allowed to homogenize. Then, 0.72 g of Ammonium Persulfate (APS, purity ≥98.0%, Sigma-Aldrich) was added, which initiates the cross linking of the two monomers of acrylamide. To expedite the curing process, 0.88 ml of *N,N,N',N'*-tetramethylethylenediamine (TEMED, ReagentPlus, 99%, Sigma-Aldrich), which serves as a catalyst, was added. Following the addition of TEMED, the solution was immediately poured into the 3D printed mold shown in Figure 1b. With the purpose of creating a mid-layer of micron-sized particles in the phantom, the solution was initially poured until it filled half the volume of the mold. After this initial section cured, micron-sized particles were sprinkled on the free surface of the gel to form a randomized speckle pattern in the mid-plane of the phantom. Subsequently, the second half of the solution was poured on top of the tracer layer to complete the layer 1 simulant.

In order to generate an interface for visualization while maintaining properties aligned with bulk brain material, we generated an outer layer with slightly different properties using a 12% (weight / volume) solution of Acrylamide in 500 ml of DI water (32) but otherwise the procedure was unchanged from that layer 1 formulation. As shown in Figure 1e, the previously synthesized layer 1 simulant was set in the middle of the layer 2 3D-printed mold. Then, the 12% PAA solution was poured in the gap between the layer 1 and layer 2 molds immediately after TEMED was added. After layer 2 was fully cured, the gelatin brain phantom was removed from the molds, obtaining a two-material phantom as shown in Figure 1f.

**Figure 2:**
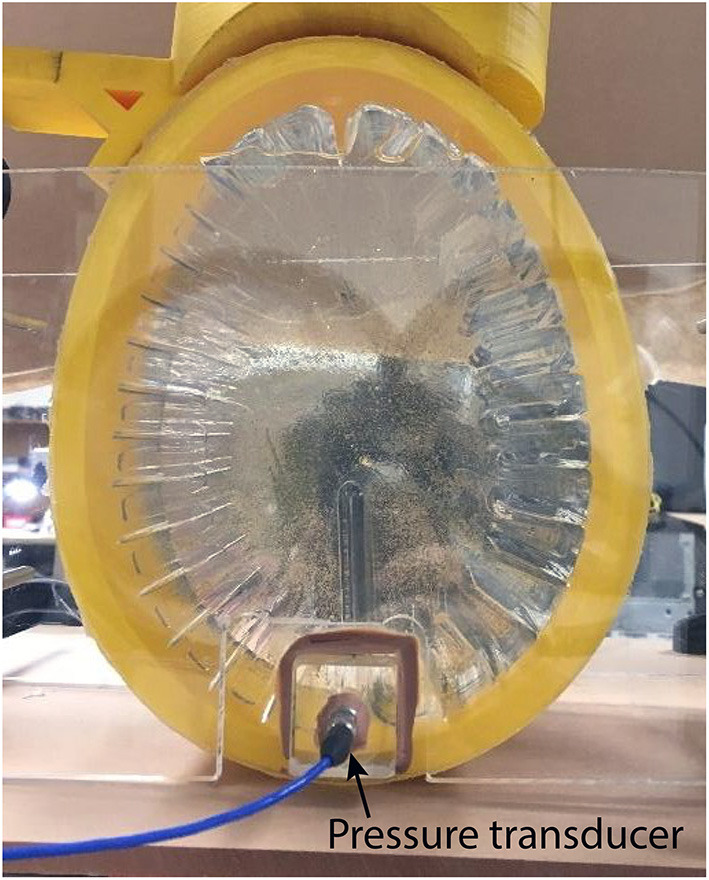
Simplified biofidelic brain phantom inside skull. The impactor is resting on the frontal region of the phantom (coup), and a pressure transducer is attached to sense the intracranial pressure around the posterior region of the phantom.

Water was used as an artificial cerebrospinal fluid (CSF), which has been widely accepted within the literature for previously performed phantom-based studies (29, 33–36). Because hydrogels are hygroscopic materials that tend to swell in aqueous environments, the phantom was pre-swelled in water for a 24-h period before its final introduction in the skull model. As shown in Figure 2, the swollen brain phantom was transferred into a 3D printed polylactic acid (PLA) skull with 50% rectilinear infill filled with DI water. Also shown in the Figure 2, a pressure sensor (PCB Piezotronics Inc., Model-113B27) was placed near the contrecoup region (approximately 1 cm anterior from intracranial countrecoup region). The test object was enclosed using clear acrylic windows.

### 2.2. Experimental Procedure

#### 2.2.1. Drop Tower

**Figure 3:**
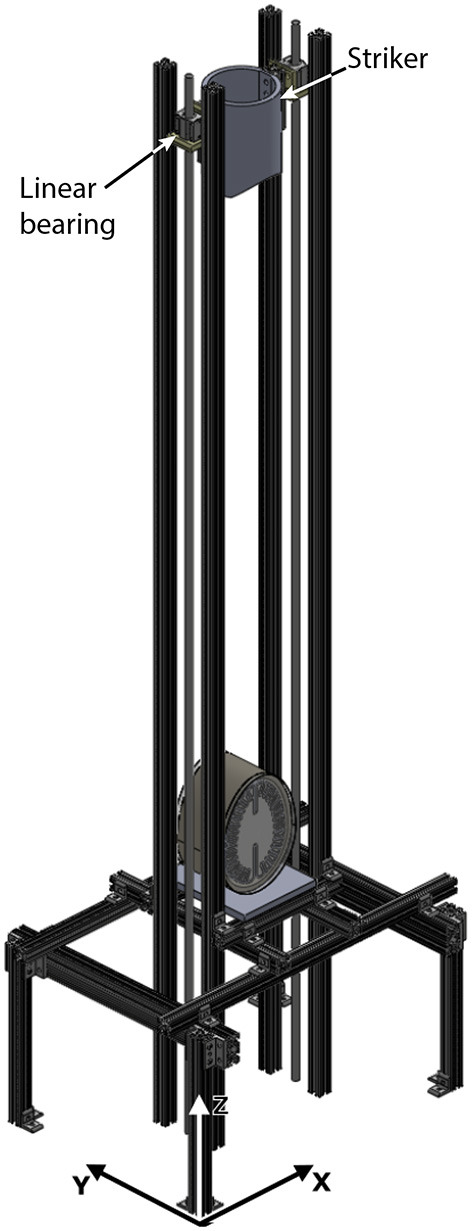
3D Representation of the Drop Tower with Impactor and Biofidelic Test Object. External dimensions: *X*-61 cm (24 in), *Y*-46 cm (18in), *Z*-183 cm (6ft).

As shown in Figure 3, a custom built drop tower was assembled out of T-slotted extruded aluminum (80/20 Inc.). The height of the drop tower was 1.829 m (6 ft.) and was installed on top of an optical table to allow for placement of the imaging system (cameras and LED lighting). The test object was placed directly in the center of the drop tower and was secured with a clear-acrylic custom frame to restrict lateral motion while allowing optical access (Figure 2).

The test object was loaded using a custom built impact striker that was 3D printed out of PLA (see Figure 3). The striker is an empty container to which 0.34 g copper balls are added up to the desired weight, with a resulting uncertainty of ±0.17 g. The bottom surface of the striker was designed with the same curvature as the frontal region of the skull simulant to distribute the load evenly upon impact (see Figure 2 for reference). This design eliminates localized loading that could lead to perforation of the skull simulant. As shown in Figure 3, the striker is guided down the drop tower using low-friction linear bearings on guide rails to ensure proper alignment upon contact with the test object.

For all experiments, the impact striker was released with initial velocity *v*_*o*_ = 0 from a height of *h* = 1.016 m (40 inches). For a free fall from this height, the effect of aerodynamic resistance on the striker is negligible (it will reduce the speed by about 0.08%). Mechanical friction is also negligible because the striker is mounted on high-efficiency linear bearings.

The striker assembly, without any copper balls, has a mass of 2.38 kg. This was the mass used for the experimental results highlighted herein. The theoretical speed, momentum, and kinetic energy carried by the striker at the instant of impact are shown in Table 1.

**Table 1:** Theoretical conditions of the striker at the instant of impact.

**Name**	**Symbol**	**Value**	
**Mass**	*m*	2.38	kg
**Speed**	*v*	4.46	m/s
**Momentum**	*p*	10.63	kg·m/s
**Kinetic energy**	*E*_*k*_	23.71	J

#### 2.2.2. Data Acquisition and Imaging

Data acquisition was performed through a custom built LabVIEW Virtual Instrument (VI) program (NI Austin, TX). The VI synchronized ultra-high-speed camera (Phantom V2512 Series) with pressure sensor data. The pressure sensor tracked the intracranial pressure (ICP) at 100 k samples per second, while the Phantom camera recorded images at 25 k frames per second at full resolution (1280 × 800 pixels), with an exposure of 20 μs per frame. This framing rate gave a time delay of 0.0389 ms between images. The field of view was 75 mm wide by 47 mm high around the posterior region of the test object, which was a magnified region of interest with magnification of 0.059 mm/pixel (in real-to-machine units). The mid-layer of particles was illuminated from the back of the test object with high-intensity LEDs, which effectively produced a speckle pattern of shadows in the images.

## 3. Experimental Results

### 3.1. Qualitative Observations

**Figure 4:**
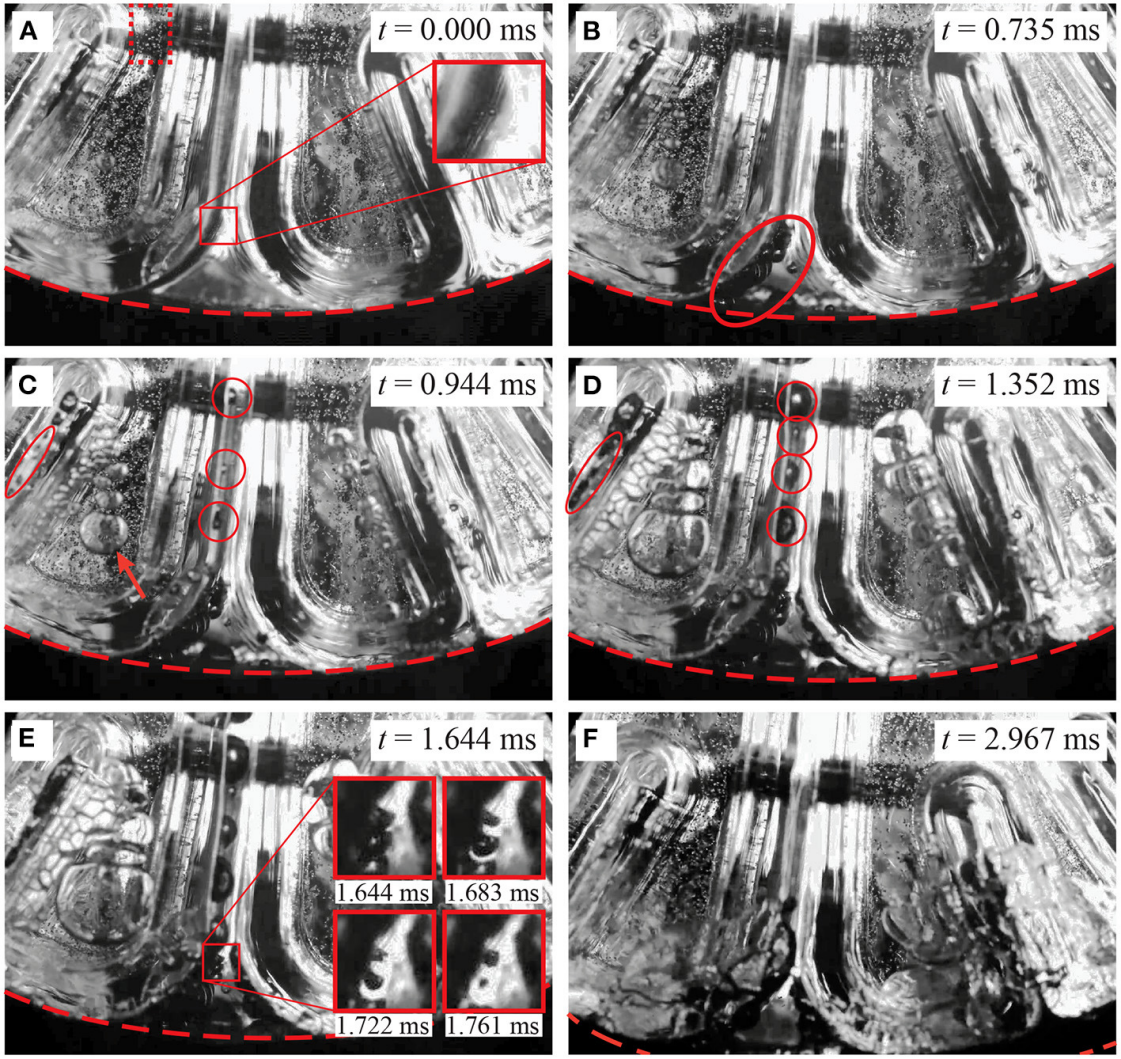
Qualitative observations in the contrecoup resulting from blunt impact. The red dashed curve demarcates the location of the inner surface of the skull. The red dotted-line rectangle in **(A)** shows the region that was observed to track the motion of the gel. **(A)** *t* = 0.000 ms: instant of impact. Two stable air bubbles are observed in the zoomed-in view. **(B)** *t* = 0.735 ms: time at which the vapor pressure of water is reached. The observed air bubbles expanded due to the pressure drop (red oval). **(C)** *t* = 0.944 ms, cavitation bubbles are observed in the sulci (red circles and oval). An arrow shows a set of air bubbles trapped in the gap between the gel and visualization window **(D)** *t* = 1.352 ms: the cavitation bubbles continue to grow while the air bubbles stay at about the same size. The cavitation bubbles appear to expand the sulci. **(E)** *t* = 1.644 ms: the cavitation bubbles over-expanded the sulci. The insets are a sequence of images in the zoomed-in view that show the process of collapsing of two cavitation bubbles. **(F)** *t* = 2.967 ms: the bulk pressure is now above the vapor pressure but below the initial intracranial pressure. All the cavitation bubbles disappeared already, while air bubbles are still present, but in the process of merging and collapsing.

**Figure 5:**
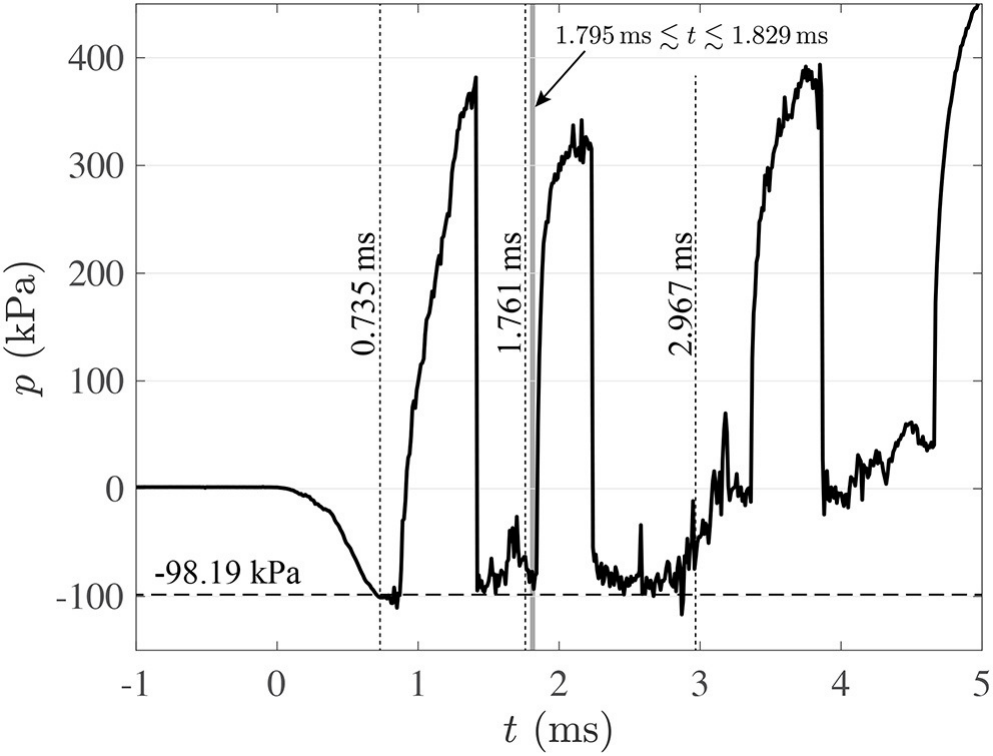
Pressure cycle at contrecoup region (–). (- -): vapor pressure at 20°C (*P*_*v*_=-98.19 kPa, relative pressure). Results for run 1.

Figure 4 shows a set of frames captured at different key instants during the first 3 ms after the blunt impact. This set of images is representative of the three different runs of this blunt-impact experiment. The following link shows a video with an overview of one of these runs: Supplementary Video 1. The following link shows a video with detailed explanations of the phenomena resulting from the blunt impact: Supplementary Video 2. Inertial cavitation was observed in all three runs, with the same qualitative behavior. Figure 4A shows the state of the TO when the impactor first made contact with the skull. At this instant, the effects of the impact have not reached down to the contrecoup region yet. Hence, Figure 4A is also representative of the state of the test object in the observed region before the impact. The small zoomed-in view inset in the figure shows two small bubbles of diameter ~0.3 mm, which were also observed prior to the impact. These are stable air bubbles, and will serve as cross-verification elements for cavitation events. This idea will become clear as these observations are further explained. As shown in Figure 4B, these two bubbles, as well as other bubbles in the same general field of view expand during the initial 0.735 ms after the impact. According to the pressure history shown in Figure 5, the bulk pressure is above the vapor pressure of the liquid (*P*_*v*_ = −98.19 kPa_relative_ at 20°C) during this initial period of time. Hence, most certainly none of these bubbles are the result of inertial cavitation, but just expanding air bubbles. The bulk pressure reaches the vapor pressure, *P*_*v*_, at time *t*≈0.735 ms. It is from this instant onward when cavitation inception would be expected to occur. In fact, at time *t* = 0.944 ms, Figure 4C shows a new set of bubbles (shown inside red circles and an oval) that appeared after the 0.735 ms time mark. Note that these new bubbles had been growing for at most 0.209 ms prior to the shown instant, and that by the 0.944 ms time mark some of them have reached sizes of roughly half the size of the initially observed air bubbles, while those air bubbles have barely increased their size during the same period of time. In fact, by *t* = 1.352 ms (Figure 4D), the new bubbles have reached sizes comparable to those of the air bubbles, while the air bubbles still have not grown significantly more beyond the size they reached at *t* = 0.735 ms. The reduced growth in the air bubbles is consistent with a constant background pressure, while the increase in size of the new bubbles under a condition of constant pressure is consistent with a phase change (evaporation). Hence, the pressure condition, rate of growth, and the fact that these new bubbles were not previously observed for *P*>*P*_*v*_ suggests that they are vapor bubbles (inertial cavitation bubbles).

For clarification, there is an arrow in Figure 4C that points at a set of relatively large bubbles. Due to their location, the reader might think that they are inside the gel, but that is not the case. These bubbles are in fact in front of the gel, trapped in the small gap between the gel and the front transparent window. This situation is verified by two conditions: first, they appear slightly out-of-focus when compared with the background particles (remember, the camera is focused at the particles in the mid-plane of the test object); second, if they were inside the gel, such large expansion would induce a large distortion in the gel itself, but that is not observed. The large size of these bubbles is the result of the fact that their confinement forces them to grow predominantly laterally. Also, any slight movement of the gel toward the window would squeeze them and make them expand laterally even further. These bubbles are more of a border effect, and could be safely disregarded for the current analysis.

As suggested above, cavitation is the product of a change of phase. As such, the pressure of the liquid/vapor mixture should remain constant at *P*_*v*_ while cavitation bubbles are forming and growing. The pressure cycle shown in Figure 5 exhibits consistency with this condition for 0.735ms≲*t*≲2.860 ms. Apart from two high-pressure excursions and some pressure fluctuations, the lowest pressure level hovers around *P*_*v*_, and the pressure tends to return to that level after the two large excursions. After *t*≈2.860 ms the pressure increases gradually and consistently and remains significantly above *P*_*v*_. Hence, *t*≈2.860 ms could be considered as the end of the cavitation period. To explain the presence of the two large pressure excursions, it is important to point out that the pressure registered by the sensor shown in Figure 1 is the result of not only the immediate vicinity of the probe, but also of pressure waves emanating from relatively distant regions of the test object.

During the cavitation period (0.735ms≲*t*≲2.860 ms), collapsing of cavitation bubbles are the most likely causes of the two large pressure excursions. This is so because the bulk pressure is ~*P*_*v*_ before each excursion, and any significant positive fluctuation in pressure sustained for enough time would make vapor bubbles collapse before any air bubble collapses. Also, the fact that the pressure returns back down to ~*P*_*v*_ after each excursion confirms that the bulk pressure is still ~*P*_*v*_ during that period of time. Note that these excursions are most likely distant local effects, because the observable vapor bubbles in Figures 4C–E are still present when the pressure excursions take place. Local fluctuations in pressure could result from the elastic response of the gelatin, which due to its geometry could present a rather complex motion (we will discuss this aspect in Section 3.2). This complex motion could plausibly induce local pressure fluctuations in the liquid. These local pressure fluctuations could trigger bubble collapsing in some isolated places. Note also that the raising edge of the first pressure excursion is less sharp than expected from cavitation bubble collapsing. This is likely due to the fact that its pressure front needs to travel through a complex geometry before reaching the pressure sensor, therefore, experiencing some attenuation.

Figure 4E shows the state of the contrecoup region at *t* = 1.644 ms. This instant is the beginning of an observable collapse of two cavitation bubbles. The zoomed-in view shows how the two bubbles have developed a dimple at the time of that capture. The other three insets show the same zoomed-in view in three successive frames (*t* = 1.6831.722and1.761ms) that show how the bubbles progressively shrink asymmetrically until collapsing. When collapsing at *t*≈1.761 ms, the bubbles produce a shock wave. This event is marked by a dotted line in Figure 5, but note that the shock wave is not detected instantly by the pressure sensor. This is so because there is a finite travel time from the origin of the shock wave to the pressure transducer. It is not clear where along the depth of the test object the observed bubbles are located, but it is reasonable to assume that they are somewhere between the mid-plane of the test object and the visualization window because they are readily visible in the field of view, unobstructed by other bubbles. Hence, their distance to the pressure transducer would be somewhere between 5 and 10 cm (the total depth of the test object is 10 cm into the page). Given that the speed of sound in water at 20°C is 1,481 m/s, the shock will take between 0.034 and 0.068 ms (or in terms of time-stamp 1.795≲*t*≲1.829 ms) to reach the pressure transducer. This interval is demarcated by a gray band in Figure 5. Note that the estimated shock arrival coincides with the raising edge of the second pressure excursion in the pressure history, confirming that this excursion is the result of bubble collapsing events. Note also that there is a noticeable positive fluctuation in pressure right before the shock formation. This fluctuation starts slightly before *t* = 1.644 ms, which is the time when the early stage of bubble collapsing was observed in Figure 4E. This observation suggests that such pressure fluctuation could have been the cause of the bubble collapsing event.

Maybe the most important aspect of the sequence from Figures 4C–E is that cavitation bubbles trapped inside the sulci seem to push the sulcal walls apart from each other. From the mechanistic point of view, this observed over-expansion of the sulci concentrates stress in the sulcal depths. This is a revealing observation because it suggests a new hypothesis for the mechanism by which Chronic Traumatic Encephalopathy is caused. Since this effect has important ramifications, it is further explored in Section 4.

Figure 4F shows the time when the bulk pressure is consistently above *P*_*v*_. The vapor bubbles disappeared completely, and the air bubbles are still present, but collapsing. In fact, the group of air bubbles initially trapped between the gel and the visualization window merged into a single large bubble and collapsed toward the bottom of the field of view. This “grand collapse” happens at around *t* = 3.360 ms, which is right after the bulk pressure reaches the initial pressure of the test object. This air bubble collapse induces a third pressure excursion. There is yet a fourth pressure excursion at around *t* = 4.660 ms, which is presumably also due to air bubble collapsing due the elevated background pressure.

### 3.2. Kinematics of Impact

**Figure 6:**
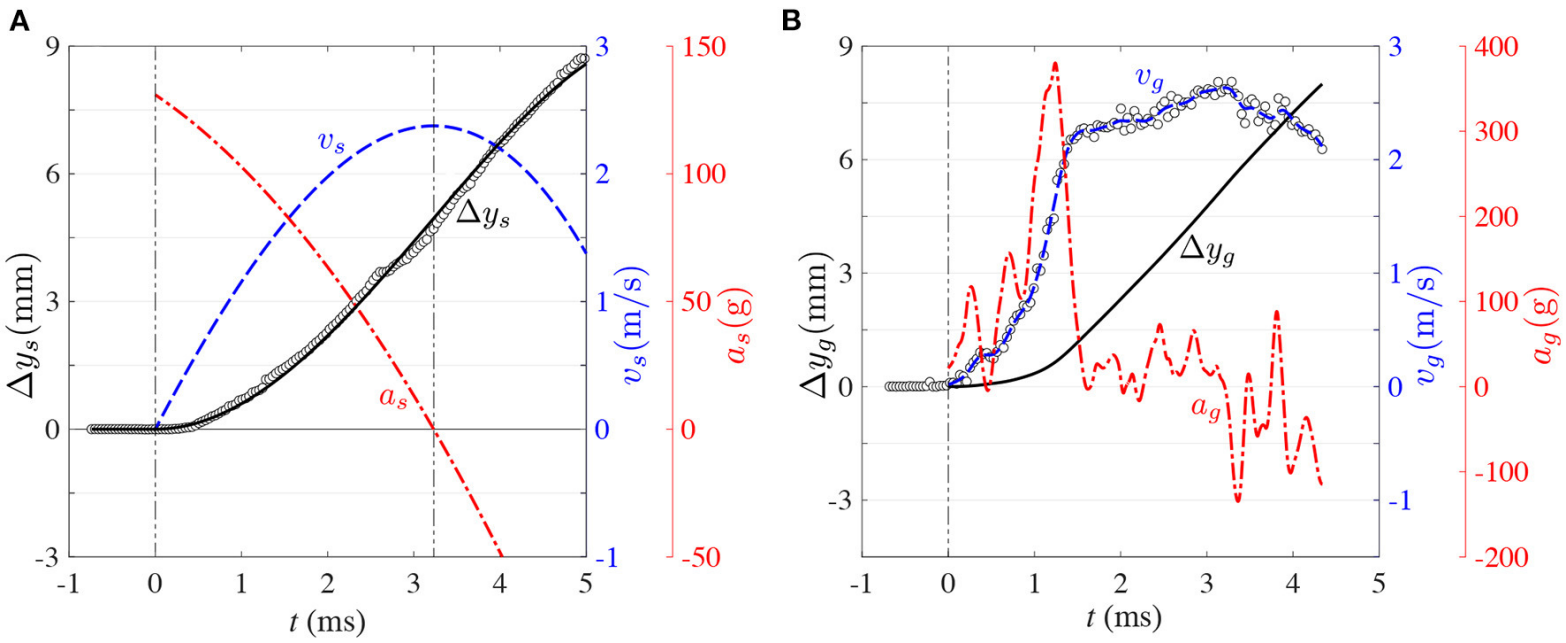
Representative kinematics of blunt impact for: **(A)** skull motion. (°): displacement, Δ*y*, based on edge-detection results. (–): displacement, Δ*y*(*t*), based on equation (1) (fitted fourth-order polynomial). (- -): speed, *v*(*t*), based on equation (2). (-·-): acceleration, *a*(*t*), based on equation (3). **(B)** gel motion. (°): speed, *v*(*t*), based on PIV. (- -): speed, *v*(*t*), based on piece-wise continuous cubic smoothing spline of PIV data. (–): displacement, Δ*y*(*t*), resulting from integrating the piece-wise continuous speed, *v*(*t*), with respect to time. (-·-): acceleration, *a*(*t*), resulting from deriving the piece-wise continuous speed, *v*(*t*), with respect to time. Results for run 1.

The sequences of high-speed images were used to determine the independent motion of the skull and gelatin. The inner surface of the skull in the contrecoup region (red dashed curve in Figures 4A–F) was tracked using digital image processing with an edge-detection algorithm [Canny edge detection, (37)]. These results are shown as open circles (°) in Figure 6A. There is some dispersion in these results because the edge of the skull is not sharply defined in the pictures (see Figure 4). Hence, the edge detection algorithm is affected by digital noise. This effect is mitigated by fitting the general equation for rigid body motion to the data. Since the skull starts its motion from rest, its initial displacement and velocity are Δ*y*_*o*_ = *v*_*o*_ = 0. With this in mind, the equations of motion corresponding to the displacement Δ*y*_s_(*t*), speed *v*_s_(*t*), and acceleration *a*_s_(*t*) of the skull, are given by:


(1) $$\begin{array}{rc} \Delta y_{\text{s}}\left(t\right) & =\frac{s}{24}t^{4}+\frac{j_{o}}{6}t^{3}+\frac{a_{o}}{2}t^{2} \end{array}$$

(2) $$\begin{array}{rc} v_{\text{s}}\left(t\right) & =\frac{s}{6}t^{3}+\frac{j_{o}}{2}t^{2}+a_{o}t \end{array}$$

(3) $$\begin{array}{rc} a_{\text{s}}\left(t\right) & =\frac{s}{2}t^{2}+j_{o}t+a_{o} \end{array}$$

Since we can determine skull displacement, Δ*y*_s_, vs. time, *t*, from our digital image analysis, we will fit equation (1) to the data. The first three coefficients of the equation have the following physical meanings: *s* is the snap (or jounce), *j*_*o*_ is the initial jerk, and *a*_*o*_ is the initial acceleration. The values of these coefficients were obtained from the data fit (Table 2), and completely define the whole set of equations of motion. Figure 6A shows a representative result (run No. 1), in which the curves resulting from the equations of motion are superimposed on the experimental data. All the runs exhibited qualitatively similar kinematics curves. Figure 6A identifies that the maximum acceleration of the skull coincides with the initial acceleration. That is: *a*_max_ = *a*_*o*_. These two values are shown in Table 2, but while *a*_*o*_ is shown in the native units of the fit (mm/ms^2^), *a*_max_ is shown in units of (*g*) for a better comparison with known scenarios of TBI-causing impacts.

**Table 2:** Coefficients for kinematics of impact and maximum acceleration.

**Run No**.	***a_o_***	***j_o_***	***s***	***a_max_***
	**(mm/ms^2^)**	**(mm/ms^3^)**	**(mm/ms^4^)**	**(g)**
1	1.231	−0.051	−0.180	125.6
2	1.295	−0.012	−0.239	132.1
3	1.283	−0.222	−0.108	131.0

The relevance of the above kinematics analysis is that this is the kind of information that conventional instruments such as accelerometers can provide. Accelerometer information nevertheless does not give a direct output of the motion experienced by the brain. What follows is the analysis of the intracranial kinematics using the motion tracers embedded in our transparent phantom. Since these tracers are distributed all across the mid-plane of the phantom, they could in principle produce a complete map of displacement and local deformation of the gel. Nevertheless, only the bulk kinematics of the phantom, and in particular the relative motion between the gel and skull, are of relevance for the current study of intracranial motion and cavitation. To this end, only the representative region of interest (ROI) enclosed in a red dotted-line rectangle in Figure 4A was used to determine the bulk motion of the gel.

Particle Image Velocimetry (PIV) was used to detect the gel's bulk motion. PIV is extensively used in fluid mechanics, the principle behind it is to compare subsequent particle images to track the motion of small groups of particles dispersed in the flow. To this end, the first image is divided in smaller overlapping sub-sets of the image called *interrogation windows*. These interrogation windows are then cross-correlated with the corresponding image sub-set in the next image (while compensating for advective motion and local deformation) to find the location of maximum coincidence with the groups of particles contained in the initial interrogation window. Depending on the domain of analysis and imaging system, PIV can produce 2D or 3D displacement fields, which can be also time-resolved or statistically independent. With the framing rate, these local displacements can be directly converted in local velocities [for more details about PIV, the reader is referred to (38)].

For the current experiments, PIV was implemented with LaVision—DaVis™. The ROI was cross-correlated recursively to gradually improve displacement estimation and reduce bias error. To this end, the interrogation window was initially defined with size 32 pixels × 32 pixels. The ROI was cross-correlated consecutively twice with this same size for an initial improved estimation of motion, and then the interrogation window size was reduced to 16 pixels × 16 pixels for two more consecutive interrogations. The calculation was compensated by rigid-body displacement and local deformation by shifting and deforming the interrogation window of the second image based on the consecutive local estimations of motion. The interrogation windows were overlapped by 50% to satisfy Nyquist criterion of sampling frequency. As a result, the resulting grid spacing between adjacent displacement vectors in image units was 8 pixels. Given that the magnification of the imaging system (in real-to-spatial units) was 0.059 mm/pixel, the spatial resolution in physical units was 0.47 mm. The ROI for PIV analysis was 128 pixels high by 120 pixels wide, resulting in a displacement vector field of size 15 rows × 14 columns, for a total of 210 vectors. The magnification in real-to-spatial units was used to express the displacement in mm; and the time delay between consecutive images, 0.0389 ms, was used to calculate the instantaneous velocity of the gel in mm/ms. To reduce sensitivity to outliers, the instantaneous bulk speed of the gel was determined as the median of each 210-vectors velocity field. These experimental results are shown as open circles in Figure 6B.

As can be seen in Figure 6B, the bulk speed of the gel is a much more complicated function of time than the bulk speed of the skull. This is so because the gel behaves as a deformable solid under the relatively high accelerations imposed in this study, as opposed to the simpler rigid body motion of the skull. Consequently, kinematics equations like the ones used for the skull (Equations 1–3) are not sufficient to describe the gel's motion. Nevertheless, the motion of the gel is constrained by the motion of the skull. Hence, while the gel moves as a deformable solid, it has to do so while tracking the motion of the skull. In consequence, the kinematic equations of the gel can be expressed as the superposition of the skull motion and deviation functions as follows:


(4) $$\begin{array}{rc} \Delta y_{\text{g}}\left(t\right) & =\Delta y_{\text{s}}\left(t\right)+\Delta y_{\text{g}}^{\prime }\left(t\right) \end{array}$$

(5) $$\begin{array}{rc} v_{\text{g}}\left(t\right) & =v_{\text{s}}\left(t\right)+v_{\text{g}}^{\prime }\left(t\right) \end{array}$$

(6) $$\begin{array}{rc} a_{\text{g}}\left(t\right) & =a_{\text{s}}\left(t\right)+a_{\text{g}}^{\prime }\left(t\right) \end{array}$$

Here, Δ*y*_s_(*t*), *v*_s_(*t*), and *a*_s_(*t*) are the displacement, velocity, and acceleration functions of the skull (from equations (1)–3), Δ*y*_g_(*t*), *v*_g_(*t*), and *a*_g_(*t*) are the displacement, velocity, and acceleration of the gel, and $\Delta y_{\text{g}}^{\prime }\left(t\right),v_{\text{g}}^{\prime }\left(t\right),$ and $a_{\text{g}}^{\prime }\left(t\right)$ are the deviations of displacement, velocity, and acceleration of the gel with respect to those of the skull. The bulk velocity of the gel, *v*_g_(*t*), obtained from PIV (open circles in Figure 6B) was modeled with an empirical function obtained via a piece-wise continuous cubic smoothing spline (blue dashed line in Figure 6B). The level of smoothing was chosen to allow meaningful calculation of temporal derivatives, while conserving the dominant trend of the data. This empirical function was integrated to obtain the bulk displacement, and differentiated to obtain the bulk acceleration. Figure 6 also shows that, as opposed to skull motion, the acceleration of the gel is highly non-monotonic and takes some time (1.242 ms) to reach its maximum. Incidentally, this maximum roughly coincides with the first pressure excursion of the pressure history (Figure 5), supporting the earlier observation that gel motion could induce localized pressure fluctuations in the surrounding liquid. And in doing so, trigger the collapse of cavitation bubbles.

**Figure 7:**
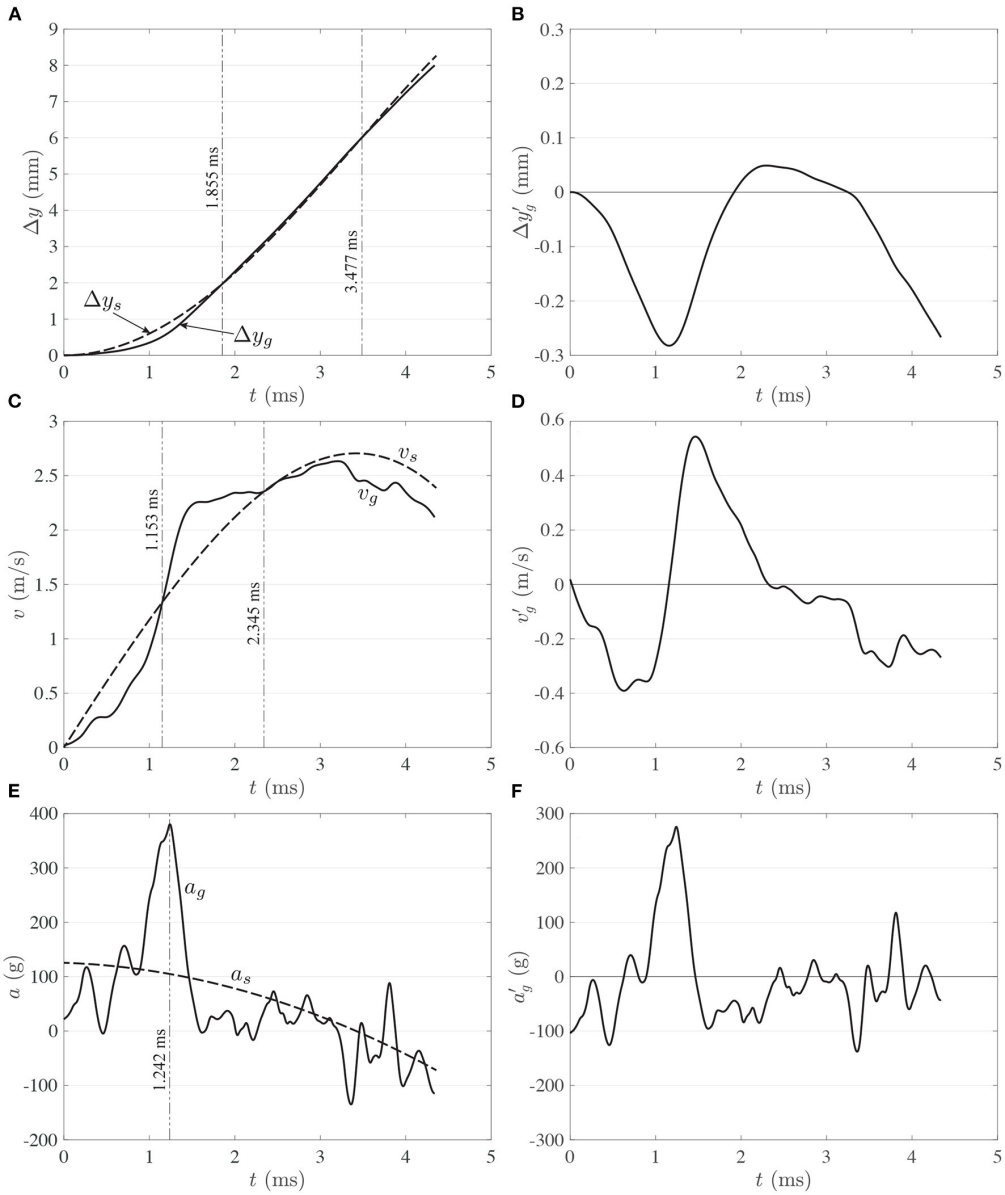
Comparison of motion between skull (- -) and gel (–) for: **(A)** displacement, **(C)** speed, and **(E)** acceleration. Gel kinematics of deviations from skull motion for: **(B)** displacement, **(D)** speed, and **(F)** acceleration. Results for run 1.

The kinematics of skull and gel are compared in Figure 7. Figures 7A,C show how the vertical displacement and velocity of the gel lag behind the displacement and velocity of the skull during the early period of the process. This is so because the motion is not transmitted instantaneously from the skull to the gel, and because the gel responds in a viscoelastic nature to the initial interaction. Due to the different response of the skull and gel to the impact load, the first 5 ms of the process could be divided in three main phases in which the displacement of the gel: 1-lags behind the skull (*t* <1.855 ms in Figure 7A), 2-overshoots the skull (1.855 ms ≤ *t* ≤ 3.477 ms in Figure 7A), and 3-lags behind the skull again (*t*>3.477 ms in Figure 7A). In phase 1, the gel basically needs to accelerate to *catch up* with the skull. During this period of time, the gel first accumulates elastic energy (predominantly in compression), which is then rapidly converted into kinetic energy as the gel accelerates. During this process, the gel reaches acceleration levels that could be three times as high as the top acceleration of the skull (Figure 7E). The gel then catches up first with the velocity of the skull (1.153 ms mark in Figure 7C) and then with its displacement (1.855 ms in Figure 7A), concluding phase 1. After these time marks, the gel overshoots the skull, both in velocity and displacement, which could presumably put it in a state of tension (this is phase 2). The acceleration then decreases sharply (Figure 7E) until the velocity reaches a plateau (Figure 7C). The velocity of the skull then catches up with the velocity of the gel (2.345 ms mark in Figure 7C), and subsequently the displacement of the skull also catches up with the displacement of the gel (3.477 ms mark in Figure 7A). At this point, the gel is presumably back in a neutral state of deformation. Then, phase 3 starts, putting the gel back again in a predominantly state of compression. A few more acceleration oscillations occur in this phase, but not comparable in magnitude to the initial spike in acceleration. Overall, phases 1 and 2 could potentially be the periods in which the most damaging events take place, at least from the point of view of material deformation and induction of collapse of cavitation bubbles.

The deviation functions are shown in Figures 7B–F. As mentioned before, rigid body motion is eliminated from gel motion to generate the deviation functions. Hence, $\Delta y_{\text{g}}^{\prime }\left(t\right),v_{\text{g}}^{\prime }\left(t\right)$, and $a_{\text{g}}^{\prime }\left(t\right)$ capture the effect of gel deformation. Without the need of a rigorous analysis, Figures 7B–F clearly show that the spectral content increases with the order of the signal. That is, $\Delta y_{\text{g}}^{\prime }\left(t\right)$ is one order lower than $v_{\text{g}}^{\prime }\left(t\right)$ because it is the result of integration, and it is clearly dominated by low frequency signal content. On the other hand, $a_{\text{g}}^{\prime }\left(t\right)$ is one order higher than $v_{\text{g}}^{\prime }\left(t\right)$ because it is the result of differentiation, and is consequently composed by high-frequency modes.

## 4. Analysis

The intra-sulcal cavitation during the experiments grossly expand the sulci (Figure 4E). The experimental results confirmed that the onset for cavitation is consistent with the theoretical value of *P*_*v*_≈−98.19 kPa (gauge), calculated for water at 20°C and at the local atmospheric pressure (101.32 MPa). The experimental onset for cavitation was determined based on the presence of the first nucleated bubbles in the field of view.

Cavitation has been theorized to be an injury mechanism - often attributed to the high focal pressures after bubble collapse (9). However, these experimental results also demonstrate the gyral deformation from bubble expansion within the sulci. From a mechanistic point of view, sulci could be considered as “notches” in a solid material. As such, their typical response to the effect of separating their walls from each other would be to concentrate strain / stress at their ends (sulcal depths). In order to semi-quantitatively evaluate the material response in the depths of the sulci, we performed 2D numerical simulations of gyral response to bubble formation.

### 4.1. Numerical Analysis

**Figure 8:**
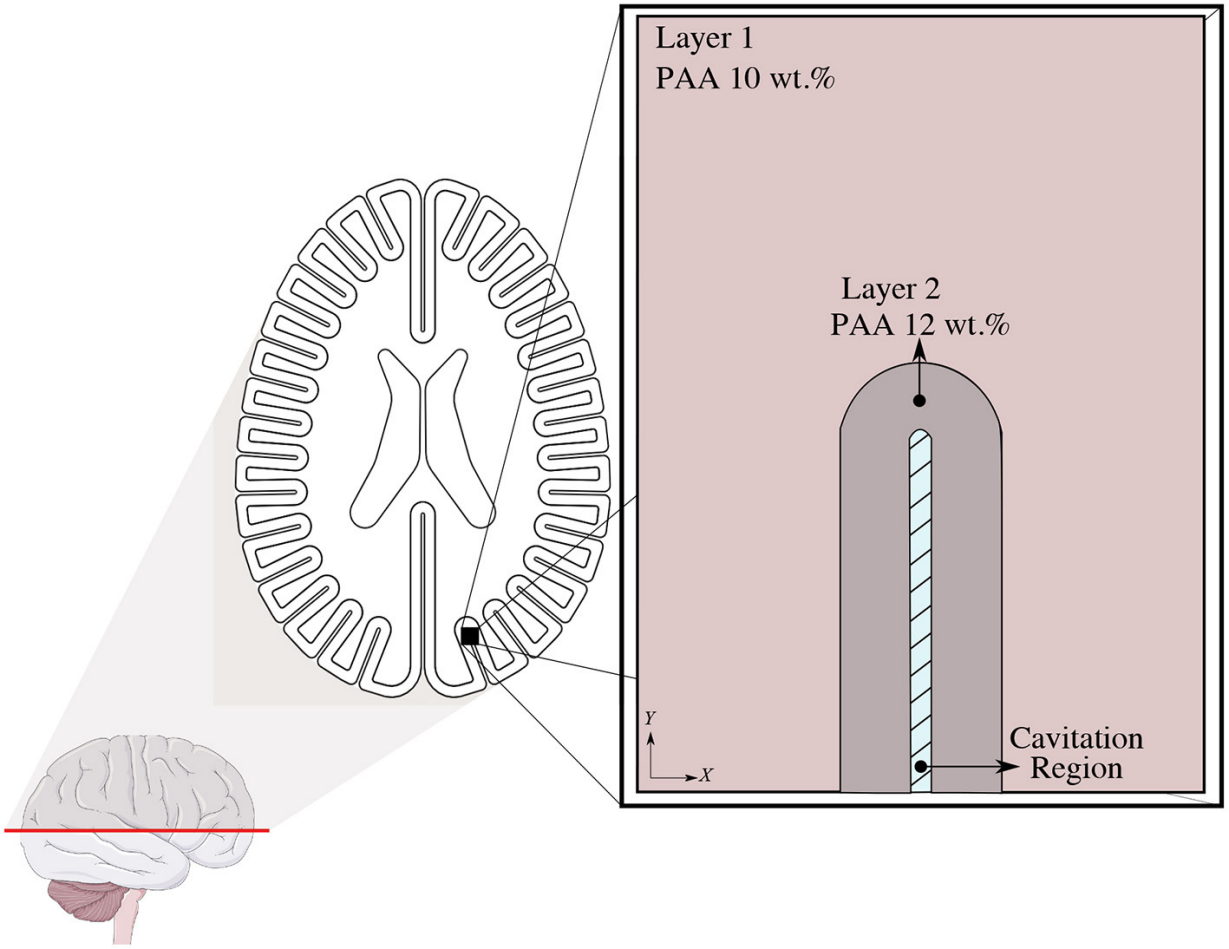
Geometric Representation of the 2D plane strain computational model domain.

Experimental observations as seen in Section 3 and discussed in Section 5 led to the creation of an elastic two-dimensional plane strain computational model to simulate the response of the brain tissue phantom to the cavitation present in the blunt-impact experiments. A numerical simulation of a single sulci was developed to elucidate the resultant tissue strain from intra-sulcal bubble expansion. The computational domain was 40 mm long by 29.32 mm wide and discretized by nearly 14,300 quadrilateral elements. The initial sulcus gap was 1.1 mm, as shown in the blue shaded area in Figure 8. The sulcus gap was also the location where the cavitation bubbles were observed from the high-speed imaging.

The numerical model consists of subdomains: an outer and inner layer and were modeled using an isotropic linear elastic material model. The material properties were matched to that of the phantom, specifically the density and high-frequency (25 Hz) shear modulus of the PAA gelatin (32). The other elastic properties (Young's and Bulk modulus) were computed by well-known elastic conversion formulas depicted by Equation (7) with the assumption of Poisson's ratio of ϑ = 0.49:


(7) $$\text{K}(\text{G,}\vartheta )=\frac{2\text{G}(1+\vartheta )}{3(1-2\vartheta )},$$

where ϑ, K, and G denote Poisson's ratio, bulk modulus, and shear modulus of the material. The material properties are tabulated in Table 3. The presented computational model was developed using COMSOL Multi-physics ® v.5.6 Solid Mechanics Module.

**Table 3:** The material parameters for different weight concentrations of PAA gelatin used in the phantom.

**Domain**	**Density** **[kg/m^**3**^]**	**Shear Modulus** **[kPa]**	**Bulk Modulus** **[kPa]**	**Poisson's Ratio**
12 wt.% PAA (Layer 1)	1037	7.27	361.47	0.49
10 wt.% PAA (Layer 2)	1056	4.19	208.45	0.49

The effect of the initiation of cavitation-induced bubbles on the surrounding tissue was characterized by a pressure boundary condition. The high-speed video images demonstrate that the range of total displacement occurring in the tissue adjacent to the cavitation bubble is between 2.30 and 2.69 mm. A parameter study was first performed to find out the required pressure level to generate the corresponding total displacement measured from the experiment. In the computational model, the deformation of tissue was modeled by applying this pressure profile. We assumed a plane strain and stationary state to simulate the deformation pattern resulted from the formation of cavitation bubbles. The roller and no-shear traction boundary conditions were defined to all outer boundaries of the domain. A displacement continuity condition was assigned to the layer 1 and layer 2 interface at the nodal level.

### 4.2. Strain/Stress Concentration at Sulcal Depths

**Figure 9:**
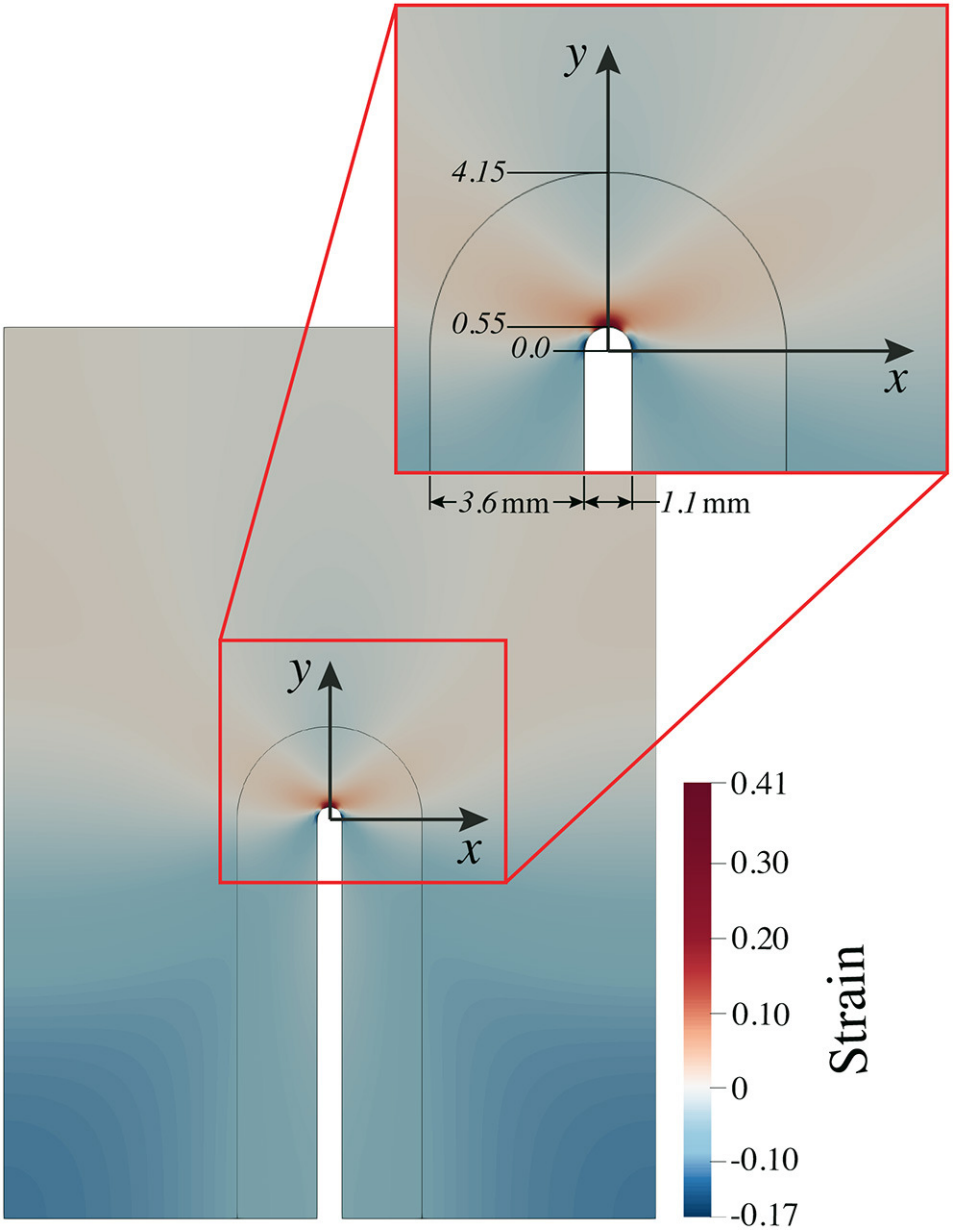
The resultant strain pattern around the sulcus due to deformation induced by expanding cavitation bubbles. Red represents tensile strain, while blue represents compressive strain.

**Figure 10:**
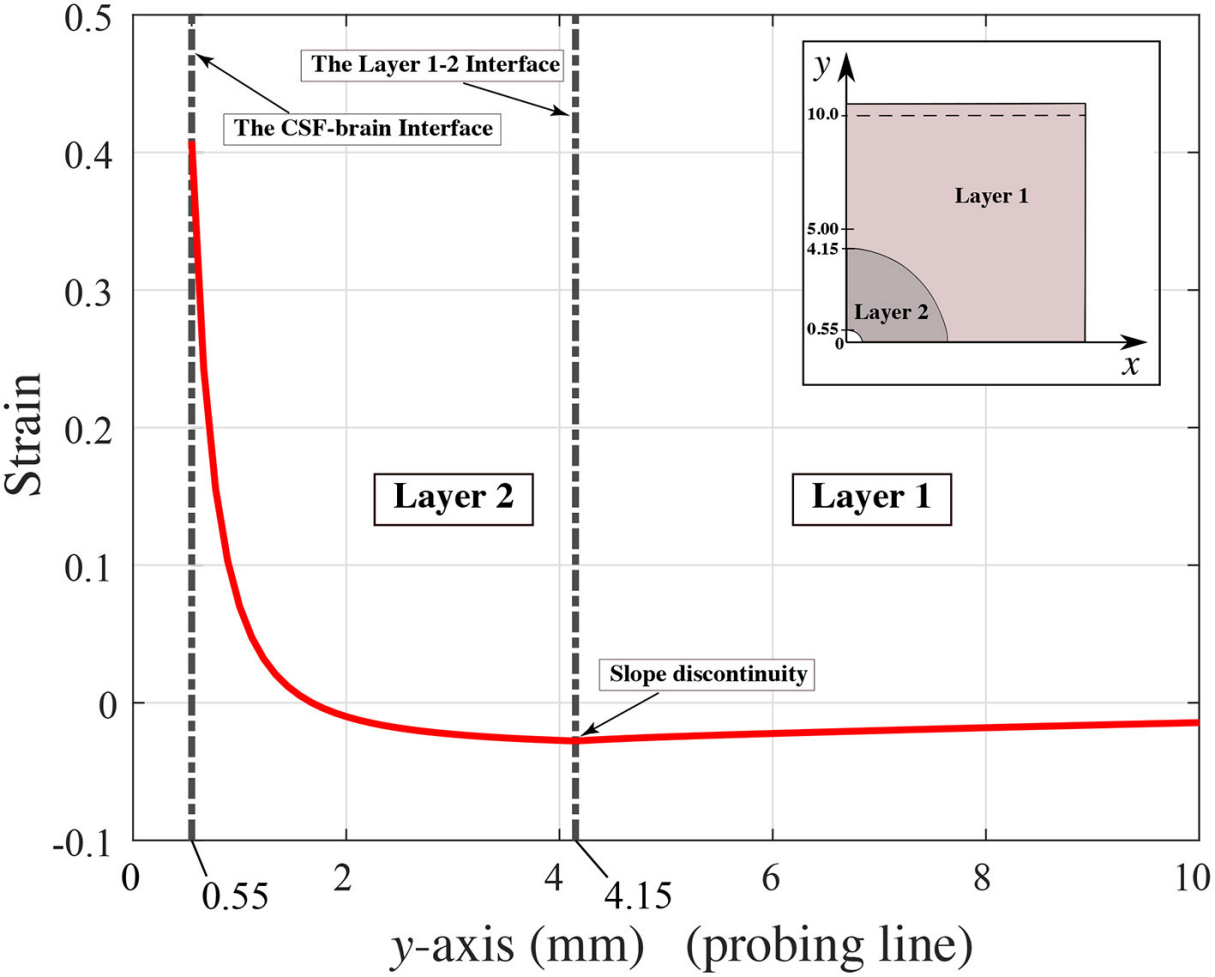
Strain distribution along the *y*−axis (probing line).

The numerical model described in Section 4.1 allowed us to detect the locations where mechanical strain is induced by sulcal expansion arising from cavitation-induced blunt trauma. The contours of strain are shown in Figure 9 to illustrate the response of the brain's soft tissue to cavitation-induced sulcal expansion. The results estimated a high tensile strain concentration at the sulcal depth due to the expansion, which is maximum at the tissue / CSF interface (0.41) right at the deepest point of the sulcus, suggesting that this is a point of stress concentration. The strain then reduces toward the bulk of the tissue. Based on Figure 10, the strain decays along the *y*−axis when traversing from the tip of the sulcus toward the bulk of the tissue. Then, the strain transitions from tensile to compressive at *y*≈1.68 mm, and keeps decaying at a lower rate until it reaches the layer 1 / layer 2 interface at *y* = 4.15 mm. At that point, the strain exhibits a slope discontinuity and reversal, relaxing asymptotically toward zero strain. Hence, this interface is also the location at which the strain attains its minimum value (−0.17), which is the maximum magnitude of compressive strain. As such, it can be considered a stress-concentrating feature, though milder than the stress-concentration at the tip of the sulcus.

A qualitative comparison between our predicted contours of tensile strains secondary to bubble expansion and the tau pathology found at the depths of the sulci have multiple similarities as has been reported in neuropathologic investigations of human subject (1, 39). Firstly, high tensile strains are located in the same location and have the same length scale ~1*mm* that is roughly symmetric between the two sides of the sulci and is of similar positioning as tauopathy observed in early CTE. The highest strain resulted from the expansion is measured as 0.41 from the location of the deepest point of the sulcus. The compressive maximum strain is 0.17 and found toward the ends of the semi-circular contour around the sulcus, as shown with dark blue in Figure 9. The compressive strain seen in the simulation results are symmetric in nature and extend distally outwards from the deepest point of the sulcus on either side. The maximum compressive strain is observed at a ±75.6° angle off of the innermost portion of the sulcus.

**Figure 11:**
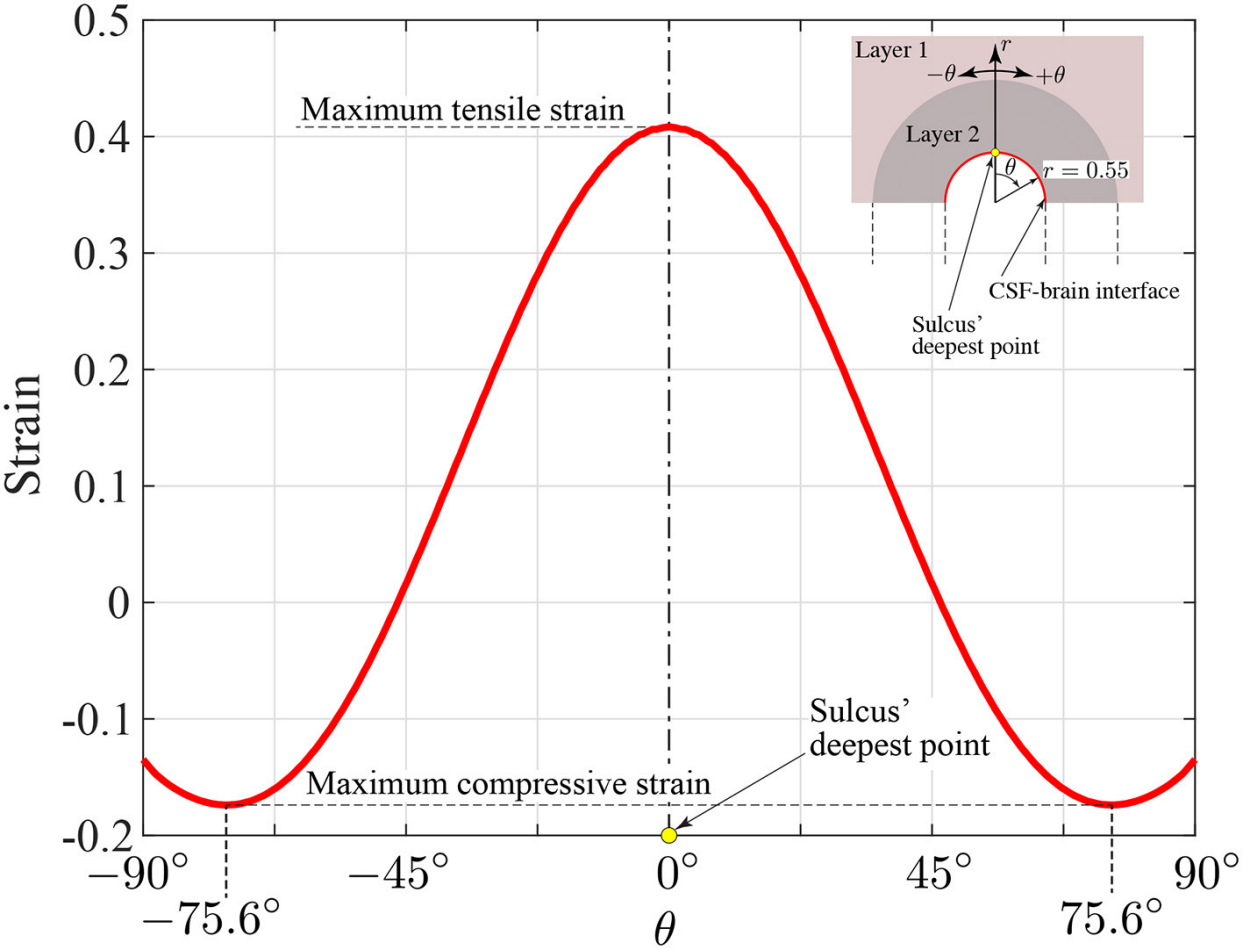
Circumferential strain distribution along the semi-circular CSF-brain interface at the sulcal depth (*r* = 0.55 mm, −90° ≤ θ ≤ 90°).

Figure 11 indicates the distribution of strain along the semi-circular CSF-brain interface at the sulcal depth. To characterize the distribution of strain along this surface, we used a polar coordinate system with radial origin at the center of the semi-circular CSF-brain interface and θ = 0° at the middle point of the arc (deepest point of sulcus). Note that, for convenience, we defined the angle as positive in the clockwise direction. Given that the sulcus is 1.1 mm wide, the radius of the CSF-brain interface (sulcal depth) is *r* = 0.55 mm. Based on this coordinate system, we recovered the strain as a function of angle along the CSF-brain interface in Figure 11. From the figure, the maximum strain (0.41) is seen at θ = 0°. The strain then reduces along the CSF-brain interface on both sides of the maximum, reaching a minimum strain value of −0.17 (maximum compressive strain) at ±75.6°.

From a mechanistic point of view, due to the anatomical structure of the sulcus and the loading conditions, the u-shaped semicircular notched edge analogy might be adopted to explain the highly localized strain pattern in the depth of the sulcus. Previous studies (40) show that there is a relation between the geometrical properties of the notch and the strain / stress concentration factor. The ratio of the depth of the sulcus to the radius of the semi-circular end is linearly proportional to the concentration factor. However, it remains uninvestigated if this relationship holds for bubble induced strain in sulci—as was found in this study.

## 5. Discussion

### 5.1. Experimental and Numerical Observations

Previous reports (41, 42) have demonstrated how differential movement between the skull and brain can produce head acceleration, which is a result of the brain lagging due to its viscoelastic properties. Rotational, but more specifically angular acceleration induces intracranial regions where the brain moves away from the skull ultimately creating regions of low pressure. These areas of low pressure can lead to contrecoup contusions (42), but if the ICP drops below the theoretical vapor-pressure threshold of the fluid, then cavitation is likely to take place.

Interestingly, the cavitation events that have been presented herein were the result of only a simple loading event. Our initial experiment utilized a flexible plate which allowed accelerations of up to 130 g. Impacts with resultant head accelerations of the same magnitude can be found in soccer, American football, and boxing (43–45). Additional experiments using a rigid plate and model human neck to support the head phantom (with lower resultant head accelerations) (not shown), also demonstrated cavitation events, however, they were less robust or generalized. The exact onset of intracranial cavitation in both our physical phantom and human subjects will require further investigations, as will correlation between onset of cavitation and clinical outcomes.

To the authors knowledge, this is the first experimental data localizing intra-sulcal cavitation and capturing the resultant response of model gyri / sulci during head trauma. Previous studies (29, 30, 34, 35) also observed cavitation as a result of loading and recent computational results also suggest intrasulcal cavitation could occur during blast exposure (46). However, the previously reported experiments supporting the cavitation mechanism either incorporated a neck-model directly, or observed the cavitation after the onset of rotational or angular acceleration. Additionally, none of the aforementioned studies incorporated models of sulci at spatial resolutions high enough to capture mechanics at the depth of the sulci. Gelatin has been used as a first-order approximation of brain tissue (29, 35), however, these were 3D solid filled ellipsoids either in a head phantom or postmortem human head surrogate. Lang et al. (30) reported on experiments with a gyrated phantom, however, the curvature of the skull and the global kinematic measurements limited observation of bubble / sulcal dynamics. We believe the incorporation of our gryi/sulci geometry into an extended 2D dimensional axial slice maintained a flat surface for clear visualization and high resolution of intrasulcal dynamics offers advantages to visualize the complex mechanics of gyri during impulsive loading.

### 5.2. Correlation With Human Pathology

During blunt impact loading of a gyrated human brain phantom we observed diffuse cavitation with subsequent expansion of the sulci during bubble formation. The resultant tissue strain from bubble formation was estimated to be almost 40% in tension in the depths of the sulci. This localization of strain maximization in the depths of the sulci has striking similarities to the distribution of neurofibrillary tangles (NFT) and tau found in the initial phases of chronic traumatic encephalopathy (1).

Chronic Traumatic Encephalopathy (CTE) is a neurodegenerative condition clinically defined by the progressive worsening of variable cognitive, affective, and motor dysfunction over the course of years. A history of repetitive head impacts (RHI) precedes the onset of symptoms with a longer and more severe exposure to RHI predicting a higher severity of pathology and clinical deficit (47). However, a history of a single head impact has also been associated with CTE (48).

Autopsy studies with age matched controls have helped to better define the condition pathologically (1). The most salient finding in early disease is the location of pathology: focal epicenters of fibrillary neuronal and astrocytic hyper-phosphorylated tau protein tangles lying in perivascular cortical tissue adjacent to the depths of the sulci along regions of the frontal lobe. TDP-43 positive neurites in the same locations can also be seen. With advancing disease, the tau pathology grows to invade broader cortical, brainstem and deep nuclear structures and generalized brain atrophy is observed macroscopically. Deep sulcal tau pathology has also been described in survivors of single TBI (48).

Like Alzheimer's Disease, CTE involves dysfunction in intracellular protein metabolism of a microtubule affiliated structure called tau, which is found in the axons of neurons (1). Traumatic forces to the brain can trigger separation of tau from the microtubule complex (49) which may lead to abnormal aggregation, misfolding, and enzymatic processing (50). Additionally, tissue deformation from high strain and strain rates has been shown to have an affiliation with neurodegeneration (51). Recent *in vitro* work also demonstrated in neurons exposed to high mechanical strain rates induce mislocalization of tau within dendritic spines in neurons (52). It has been hypothesized that the pathologic tau can act as a contagion for neuron to neuron spread, similar to a prion-like process (53) or by calcium dysregulation in the receptive neuron (54). Impulsive loading of the brain appear to activate neuroinflammatory cascades within the cortex (55) and emerging evidence also implicated neuro-inflammatory pathways in the disease, with extracellular hyperphosphorylated tau activating microglia and astrocytes, which in turn upregulate inflammatory cytokines and chemokines and a cycle of ongoing inflammation and tau phosphorylation follows (56). Furthermore, many of the pathways linked to the development of NFTs evidence that they can be initiated by either trauma or blast exposure (57). Likewise, elevated CD68 density, a marker of chronic phagocytic / inflammatory activity, was found to have an affiliation with elevated tau pathology in the dorsal lateral frontal cortex of individuals with a history of RHI (58).

Strain localization within sulci does not require cavitation (6, 7). However, there are significant differences between the strains between cavitation induced sulcal strain vs. the strain that would be encountered during TBI without cavitation. Firstly, we estimate strains in depths of sulci as high as 40%, which is approximately double the strains reported in (7) and those used experimentally in (52). In our model, bubble expansion occurred over ~1*ms* with induced strains of 40%, thus maximal strain rates would be on the order of 400 *s*^−1^ which also would be intuitively higher than the strain rates induced by the lower amplitudes and shorter time duration of non-cavitation induced strain. Thus, cavitation inception could represent a dramatic and perhaps extreme form of sulcal injury which may initiate a different pathology than lower amplitude (and non-cavitating) impacts.

Furthermore, recent experimental studies using cast models of human brain material and craniums also demonstrated cavitation near the contrecoup for impacts of similar magnitudes (30). Thus, there is developing evidence that mechanical strain leads to phosphorylation tau dysregulation (52) and that strain focusing will occur with the depths of the sulci with or without sulcal cavitation. However, further investigations are needed to identify the conditions necessary to induce the progressive formation of NFT's. It is possible that cavitation's resultant higher strains and strain rates within the depths of the sulci may have unique pathophysiologic outcomes over strain concentration during non-cavitating head impacts. It is also possible that the higher strain and strain rates from intrasulcal cavitation represent a more severe instance of sulcal strain focusing that would be expected during traumatic brain injury in a gyrated brain.

### 5.3. Limitations

There are multiple limitations to this study.

Firstly, the human head phantom employed is an idealization of the human brain. Although it captures multiple key features of a human head—volume, size, matching of length scales for sulci and gyri—it does not have detailed anatomy to represent any specific human skull or even differentiate between the different lobes of the human brain or the non-uniform anatomy of the human brain. The human skull varies greatly in thickness with different susceptibilities for fracture and transmission of forces intracranially as a function of impact location. The lack of any of these anatomic features does limit the ability to make specific predictions about specific types of impacts leading to specific patterns of cavitation (and possible injury). Ventricular pathology is also apparent in CTE cases and further modifications of our phantom will include model ventricles. Although there could be interesting dynamics within the ventricles and the periventricular region, given the spatial separation between the ventricles and sulcal spaces, it is not anticipated that intra-sulcal cavitation would be greatly altered by the presence of ventricles. Additionally, in humans, sulci are not straight. The introduction of curvature or bends to the sulci would lead to strain concentration near regions of high curvature, however, computer simulations of higher fidelity brain models still predict the maximum strains (in non-cavitating models) at the depths of the sulci (6). However, what is unclear is whether curvature or bends in sulci could modify the likelihood of cavitation inception and thus is a reasonable hypothesis to test experimentally. Lastly, an important, yet missing anatomic model in our phantom is vasculature. Arterial vasculature resides in the same subarachnoid space where cavitation is hypothesized to exist and thus could be directly injured from a cavitation event. The presence of vascular structures within the CSF space further confines fluid and this confinement could increase likelihood of cavitation—as has been suggested in computational simulations of blast injury (46). However, the mechanisms of cavitation within confined fluid spaces remain complex and will require dedicated studies.

Despite the limitations our phantom anatomy imposes, its simplicity allows for the generalization and intuitive understanding of a possible novel mechanism of brain injury during impact—specifically, the inception, expansion, and collapse of bubbles deep into the sulci, which ultimately leads to significant intrasulcal strains. Another limitation is that our phantom has yet to be validated against *in vivo* humans to verify that it captures the gross motion of human brains under linear acceleration. This work is ongoing, however, the bulk material properties of our phantom have been previously tested and have matched shear properties to human brain tissue (32). Given the bulk matching of properties and length scales between a human head and this phantom, phenomenology found in this model remains likely representative of intracranial mechanics during head impacts. Our current material models are also homogenous and do not reflect the complex anisotropy of the human brain because of connective tissue, white matter tracts, or vasculature. Although these limitations could alter the long time behavior of the phantoms and larger scale / bulk motion—it should not be anticipated that this simplification would alter the sub-millisecond inception of cavitation bubbles. Anisotropy of tissues could alter the resultant expansion of sulci; however, most of the mechanical strain from bubble expansion occurs within a fraction of a millimeter of the surface—which is gray matter that is often assumed to be grossly homogeneous. Thus, a lack of anisotropy would also not be anticipated to significantly alter the magnitude of intrasulcal strain.

A second limitation of this work is accounting for the presence of microbubbles within our phantom. Despite using a deionized CSF model and manual manipulation of gyrated phantom to remove bubbles the high speed imaging and video demonstrates the presence of multiple small bubbles out of the focal plane prior to impact. Although within the sulci of interest we did not visualize any pre-existing bubbles, it is also possible that there concurrently existed multiple microscopic bubbles within the sulci which expanded during the negative pressure phase of the impact—which would then be responsible for the observed behavior of sulcal expansion from bubbles. However, the careful qualitative observations and correlation of bubble dynamics with intracranial pressure are indicative that a portion of the intrasulcal bubbles were formed only during periods of pressure below vapor pressure—strongly supporting the presence of cavitation within the sulci. However, even though there was a presence of pre-existing bubbles within our phantom (which expanded prior to intracranial pressure being below vapor pressure) we do not believe this negates sulcal expansion from cavitation as a hypothesis injury within depths of the sulci. Firstly, other models in both blast and blunt exposures have demonstrated cavitation (25) and computational models predict cavitation from impacts (7). Figure 4 also demonstrates that bubble growth initiated near the vapor pressure of our CSF surrogate. Secondly, the presence of microscopic bubbles within CSF or biologic tissue has been hypothesized to exist *in vivo* as even nanometer scale bubbles can be stabilized within low concentration NaCl fluid (such as CSF) or near fluid-solid interfaces (59). Thus, the presence of remnant microbubbles within the depths of the sulci from imperfect degassing of the CSF surrogate does not necessarily negate the validity of our generated hypothesis. Future work, beyond the scope of this manuscript, will need to both quantify the presence and degree of existing micro/nano bubbles intracranially or visualize and quantify intrasulcal cavitation (in the resultant gyral strain) in *in vivo*, gyrated animal models.

Another key limitation is the lack of direct neuropathologic correlation to the strains measured within the sulci. The current biofidelic brain phantom does not have any ability to model physiological changes associated with TBI or intracranial cavitation. Further work is necessary to quantify the expected pathology and clinical correlation from intrasulcal cavitation—be it within *in vitro* models or via detection of intrasulcal measurements in gyrated *in vivo* models.

A additional limitation of the work is the somewhat artificial loading scenario. Most head impacts yield both linear accelerations and angular accelerations. Video and accelerometer data from athletes (44, 60) show the presence of both linear and angular accelerations, which would induce further complexities in the resultant intracranial motion. Multiple hypotheses exist suggesting angular rotation is responsible for clinical concussions and comprehensive models of brain injury will need to account for angular accelerations. However, in order to gain maximal insight into the multiple mechanical phenomena occurring during a head impact, isolating linear acceleration is an important step in understanding the intracranial kinematics during blunt loading. Furthermore, this current setup does allow for highly reproducible and easily tunable blunt impacts as is desired in both blast and blunt TBI studies (61).

## 6. Conclusions

Using a novel gyrated human head phantom, undergoing realistic impacts, we visualized diffuse cavitation within cerebrospinal fluid. This cavitation lead to sulcal expansion and high strains and strain rates at the depths of the sulci—which matches qualitatively with the tauopathy / neurofibrillary tangles pattern found in early chronic traumatic encephalopathy. This work further supports the strain focusing hypothesis as a possible initiating event for CTE. Further work is needed to characterize if the presence of intrasulcal cavitation leads to unique pathology from traumatic brain injury vs. a more extreme case of strain focusing expected within a gyrated brain following a mechanical insult.

It remains to be seen how cavitation events are affected by modifications in the parameter space of this experiment. That is, by changing aspects such as: head phantom's orientation with respect to impact, momentum and energy at impact (controlled by weight and height of load), presence of additional anatomical structures (e.g., blood vessels, ventricles, falx cerebri), and/or modifications in phantom's design (e.g., size, representative section, curved calvarium, curved gyri). Since these aspects are too extensive to include in this manuscript, they will be addressed in future studies.

## Data Availability Statement

Any party interested in the raw data that support the conclusions of this article should contact the corresponding author Adam M. Willis.

## Author Contributions

JK, AW, and RM-A contributed conception and design of the study. SV acquired data and provided technical assistance. MT created the original brain molds. JK and BD-M performed the post analysis and wrote the first draft of the manuscript. AY and TP were responsible for the computational simulations. JK, BD-M, AY, JV, and RM-A wrote different sections of the manuscript. JK, BD-M, AW, AY, SV, and RM-A contributed to manuscript revision, proofread, and approved the submitted version.

## Funding

This material is based on research sponsored by the U.S. Air Force under agreement number FA8650-18-2-6880. This work was also sponsored in part by Michigan State University.

## Author Disclaimer

The U.S. Government is authorized to reproduce and distribute reprints for Governmental purposes notwithstanding any copyright notation thereon. The view(s) expressed herein are those of the author(s) and do not reflect the official policy or position of Brooke Army Medical Center, the U.S. Army Medical Department, the U.S. Army Office of the Surgeon General, the Department of the Army, the Department of the Air Force, or the Department of Defense or the U.S. Government. Mention of trade names, commercial products, or organizations does not imply endorsement by the U.S. Government. Some of the authors are military service members or federal/contracted employees of the United States Government.

## Conflict of Interest

JK, AW, MT, and RM-A are listed as inventors in a patent application for the Biofidelic Model of the Human Brain.

The remaining authors declare that the research was conducted in the absence of any commercial or financial relationships that could be construed as a potential conflict of interest.

## Publisher's Note

All claims expressed in this article are solely those of the authors and do not necessarily represent those of their affiliated organizations, or those of the publisher, the editors and the reviewers. Any product that may be evaluated in this article, or claim that may be made by its manufacturer, is not guaranteed or endorsed by the publisher.
